# The PKHD1 gene inhibits tumor proliferation and invasion in intrahepatic cholangiocarcinoma by activating the Notch pathway

**DOI:** 10.7150/ijms.95964

**Published:** 2024-10-14

**Authors:** Tianyu Shang, Xiaoning Chen, Hanxin Xue, Yinlian Wu, Su Lin, Yueyong Zhu

**Affiliations:** 1 Department of Hepatology, The First Affiliated Hospital, Fujian Medical University, Fuzhou, Fujian, China.; 2 Hepatology Research Institute, Fujian Clinical Research Center for Liver and Intestinal Diseases, Fuzhou, China.; 3 National Reginal Medical Center, Binhai Campus of the First Affiliated Hospital, Fuzhou, China.; 4 Guoke Ningbo Life Science and Health Industry Research Institute, Ningbo, China.; 5 Ningbo No.2 Hospital, Ningbo, China.

**Keywords:** Intrahepatic cholangiocarcinoma, Invasion, Notch pathway, PKHD1

## Abstract

**Background:** Intrahepatic cholangiocarcinoma (ICC), one type of highly malignant tumor, has a poor prognosis. However, the specific role of the polycystic kidney and hepatic disease 1 (PKHD1) gene in ICC has not yet been evaluated. This study aimed to investigate the potential function and mechanism of the PKHD1 gene in ICC.

**Methods:** Quantitative real-time PCR was applied to detect the expression of PKHD1 mRNA in human ICC and adjacent normal tissues. CRISPR/Cas9 technique was used to construct PKHD1 partially knockout (PKHD1-/+) ICC cell lines. In the vitro study, the effects of PKHD1 on the malignant biological behavior of ICC cells were examined by Edu, RTCA, migration, and invasion assays. The expression levels of proteins were detected using western blotting, immunohistochemistry, and flow cytometry. Furthermore, DAPT, an antagonist of the Notch1 signaling pathway, was used in the rescue experiment *in vitro*.

**Results:** Compared with normal tissues, PKHD1 mRNA expression was significantly down-regulated in human cholangiocarcinoma tissues (*P*<0.001). At the same time, the expressions of Notch pathway-related proteins were dramatically increased in PKHD1(-/+) ICC cells (*P*<0.001). Moreover, tumor proliferation, migration, and invasion were promoted in loss-of-function experiments *in vitro* and *in vivo*, which was partially reversed by DAPT.

**Conclusions:** PKHD1 inhibits the proliferation, migration, and invasion of ICC, and the Notch pathway may be the downstream mechanism of the negative regulatory effect of PKHD1 during the progression of ICC.

## Introduction

Intrahepatic cholangiocarcinoma (ICC) is a highly malignant tumor originating from the epithelium of the bile duct. It is the second most common primary liver cancer with rising global morbidity and mortality in recent years^1^, ^2^. Due to the occult onset and the lack of effective early screening methods, most ICC patients are diagnosed in the advanced stage and only 20%-30% of them are candidates for radical surgery at the time of diagnosis^3^. Despite the rapid development of medical science and technology in recent years, the mortality rates of ICC have been reported to be stable in most countries, with a 5-year overall survival rate remaining around 9%^3^, ^4^. Even after radical surgery, the recurrence rate after surgery is as high as 40%-80%, with a 5-year survival rate of 10%-30%^5^.

Established risk factors for ICC include fibropolycystic disease and some genetic conditions, including cystic fibrosis, and biliary papillomatosis^6^, ^7^. As the causative gene of autosomal recessive polycystic kidney disease, polycystic kidney and hepatic disease 1 (PKHD1) gene encodes the fibrocystin/polyductin (FPC) protein, which is expressed in not only the kidneys but also bile ducts^8^. Previous studies suggested that FPC plays an important role in the proliferation and differentiation of biliary epithelial cells, and the defect of FPC causes abnormal development of biliary epithelial cells and the biliary tract^9^. Moreover, an increased prevalence of liver neoplasms has been reported in patients with polycystic kidney disease, which indicated that the PKHD1 gene may be related to the occurrence of liver cancer, especially ICC^10^. The Notch receptor, which is located on the surface of bile duct epithelial cells along with the FPC protein^11^, is an important protein for the development of intrahepatic bile duct cells and the formation of the three-dimensional structure of the bile ducts. It also participates in the process of liver injury repair and the development of liver tumors^12^, ^13^. In recent years, the concern of the PKHD1 gene and Notch signaling pathway in liver disease has increased. However, the role and potential mechanism of PKHD1 in ICC have never been investigated to date.

Therefore, the objective of this study is to investigate the potential function and mechanism of the PKHD1 gene and the Notch signaling pathway in ICC.

## 2. Materials and Methods

### 2.1. Human tissues

ICC tissues and adjacent normal tissues were collected from the First Affiliated Hospital of Fujian Medical University from September 2017 to September 2018. The protocol was approved by the Ethics Committee of Fujian Medical University conforming to the ethical guidelines of the Declaration of Helsinki. All subjects provided written informed consent for using human materials in this study.

### 2.2. RNA isolation and quantitative real-time PCR

Tissue RNA was extracted using TransZol Up Plus RNA Kit (Transgene, ER501). First-strand cDNA was synthesized from total RNA using oligo dT primers using Transcriptor First Strand cDNA Synthesis Kit (Roche, 04896866001). Quantitative real-time PCR was performed using SYBR Green on ABI 7500 System (Applied Biosystems), primer sequences as follows: PKHD1: forward primer (5'-TCCAAACGCCGAGAATCACA-3') and reverse primer (5'-TTCCTCTCGGACAATGTGGC-3'); GAPDH: forward primer (5'-GAAAGCCTGCCGGTGACTAA-3') and reverse primer (5'-GCATCACCCGGAGGAGAAA-3').

### 2.3. CRISPR/Cas9 plasmid and design

PKHD1 genomic sequence (NM_138694) was submitted to the online gRNA design website (http://crispr.mit.edu). Then sgRNAs were screened by Cas-Offinder and three of those with fewer mismatches were chosen (*Supplemental Table 1*). The construction of recombinant plasmid and the packaging of lentivirus were completed by Jikai Biotech (Shanghai, China) according to the above sequences.

### 2.4. Cell culture and transfection

Human ICC cell lines HCCC-9810, RBE, IHC-ST1, and HuCCT1 were obtained from the Cell Bank of the Chinese Academy of Sciences (Shanghai, China). These human ICC cells were grown in DMEM medium (Gibco, Carlsbad, CA, US) containing 10% fetal bovine serum (FBS; Gibco) and incubated at 37 ℃ with 5% CO_2_. Transfection was performed in RBE cell lines using polybrene (GeneChem, Shanghai, China) according to the manufacturer's instructions. Analyses were conducted at 48 h after being transfected.

### 2.5. Flow cytometry

To detect the knockout of PKHD1 protein in RBE and HCCC-9810 cell lines, flow cytometry was conducted using a Thermo Fisher flow cytometer (Waltham, MA, US). The PKHD1 antibody was purchased from Abcam Bioscience (Cambridge, UK), and data analysis was performed using FlowJo software (Becton Dickinson, Franklin Lake, NJ, US).

### 2.6. Proliferation assay

Cell proliferation was determined by 5-Ethynyl-2'-deoxyuridine assay (EdU; Beyotime, Haimen, Jiangsu, China) and RTCA (Real-Time Cellular Analysis). Assays were performed according to the manufacturer's instructions.

### 2.7. Migration and invasion analysis

Migration was detected by scratch and Transwell assays. In the scratch assay, the PKHD1(-/+) RBE cells were cultured in 6-well plates until covering 90% of each well. Micropipette tips were applied to create scratches in the wells. The scratch was observed and photographed at 0h, 12h, and 24h. For the Transwell assay, the transfected RBE and HCCC-9810 cells were placed in top chambers (Corning, NY, United States) with a serum-free medium. A culturing medium containing 10% FBS was added into the bottom chambers as an attractive substance. After incubation for 24 hours, the cells in lower chambers were fixed, stained, and counted manually. Invasion was also detected by Transwell assay and 50µl diluted matrix gel (Corning, NY, United States) was added to the upper chamber one day before.

### 2.8. Colony Formation Assay

Cells were seeded in 6-well plates at low density (500 cells/well) and cultured for 21 days. Cell colonies were fixed in 4% paraformaldehyde for 15 min, and 1 ml 0.1% crystal violet was added (Sigma, USA) to each well for 30 min at room temperature for visualization.

### 2.9. Western blotting

Cells or tissues were isolated and denatured in an SDS buffer for total proteins. Proteins were quantified using BCA Assay Kit (Thermo Pierce, MA, USA), and then separated by 10% SDS-PAGE and transferred to PVDF membranes. The membranes were blocked with TBST containing 5% BSA at room temperature for 1 h and then incubated overnight at 4 °C with anti-Notch1 (1:500, Abcam, Cambridge, UK), anti-NICD, anti-Snail+slug, anti-MMP9 and anti-GAPDH (1:1000, Abcam). After washing, the membrane was incubated with HRP-conjugated goat anti-rabbit/mouse IgG (Abcam) at room temperature for 1 h, then washed three times in TBST (Solarbio, Beijing, China) and visualized using ECL reagent (Thermo).

### 2.10. Tumor formation in nude mice

The PKHD1 knockdown RBE and normal control cells were trypsinized and resuspended in the medium at a concentration of 3×10^7^ cells/ml. Then, these cells (0.2 ml) were injected subcutaneously into the left armpits of 5-week-old male BALB/c nude mice respectively (SLAC, Shanghai, China). The subcutaneously growing tumors were evaluated every two days after transplantation. The mice were sacrificed after 4 weeks and the weights of the subcutaneous tumors were recorded. The tissues were embedded in paraffin, sectioned, and then stained to determine the protein expression via immunohistochemistry. The trial protocol was approved by the Experimental Animal Ethics Committee of Fujian Medical University. All surgery was performed under sodium pentobarbital anesthesia, and all efforts were made to minimize suffering.

### 2.11. Immunohistochemistry

The tissues were fixed in formalin and then embedded in paraffin. The blocks were cut into 4-mm thick, deparaffinized, and rehydrated. After blocking with H2O2 (3%), the samples were incubated with anti-PKHD1 (1:200, Abcam), anti-snail+slug (1:1000, Abcam) and anti-MMP9 (1:200, Abcam) at 4 °C overnight. After washing with phosphate-buffered saline, the samples were incubated with the secondary antibody (Alxea flour 647, Thermo) for 1 h at room temperature. Then, the samples were stained using DAB (Sigma-Aldrich, St. Louis, MO, USA), and re-stained by hematoxylin.

### 2.12. Statistical analysis

Cell experiments were performed at least in triplicate. Data were statistically analyzed using SPSS software (version 19.0, Chicago, IL, USA). Quantitative data were analyzed by Student's t-test, and the results are expressed as the mean ± SD. *P* <0.05 was considered statistically significant.

## 3. Results

### 3.1. The expression of PKHD1 is decreased in human cholangiocarcinoma tissues and varies in different ICC cells

**Figure 1:**
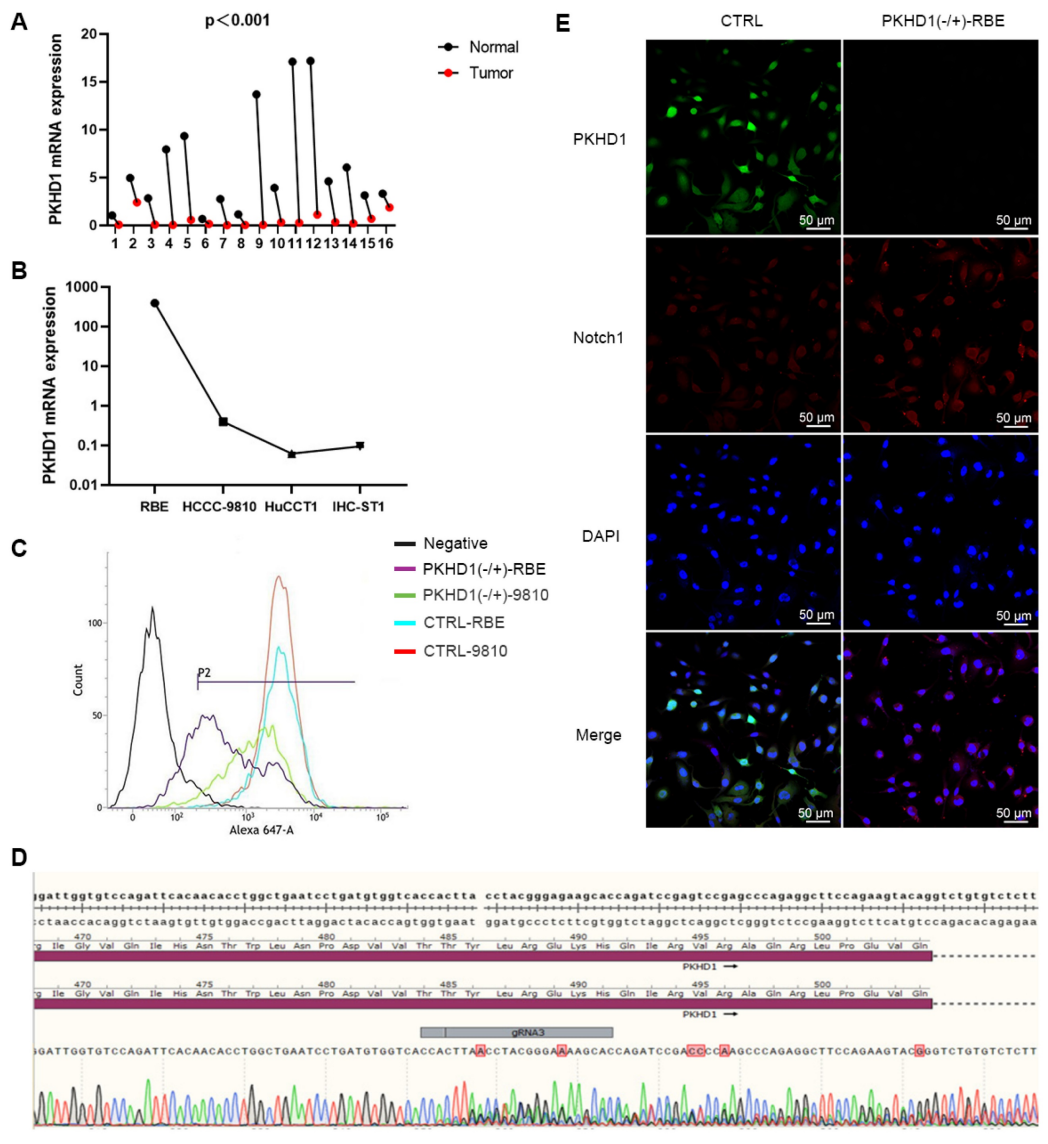
The PKHD1 mRNA detection, positive cell lines screening, and post-transfection verification. (A) The relative expression of PKHD1 mRNA in tumor and adjacent normal tissues of patients with ICC (***, *P* < 0.001); (B) The relative expression of PKHD1 mRNA in different ICC cell lines; (C) Confirmation of PKHD1 partial knockout by flow cytometry; (D) Sequencing verification of Cas9-RBE stable transgenic strains; (E) Immunofluorescence colocalization of PKHD1 and Notch1 protein.

ICC and adjacent normal tissues from 16 patients with cholangiocarcinoma were collected. Then, the expression levels of the PKHD1 gene were detected using qRT-PCR. The expression of the PKHD1 mRNA in ICC tissues was dramatically decreased than that in normal tissues (*Figure 1A*, *P*<0.0001). Among the four different ICC cell lines (RBE, HCCC-9810, IHC-ST1, and HuCCT1), PKHD1 expression was highest in RBE cells and lowest in HuCCT1 cells (*Figure 1B*).

### 3.2. Identification of PKHD1 knockdown ICC cell lines by flow cytometry

RBE and HCCC-9810 monoclonals did not amplify during transfection, therefore, mixed clones were selected to conduct the sequencing verification and identification of PKHD1 knockdown (*Figure 1C-D*). Positive RBE clones were selected for follow-up experiments, named PKHD1(-/+)-RBE/9810. Wild-type clones and clones transfected with empty vectors were named WT and CTRL, respectively.

### 3.3. Knockdown of PKHD1 enhances proliferation, migration, and invasion of ICC cells

**Figure 2:**
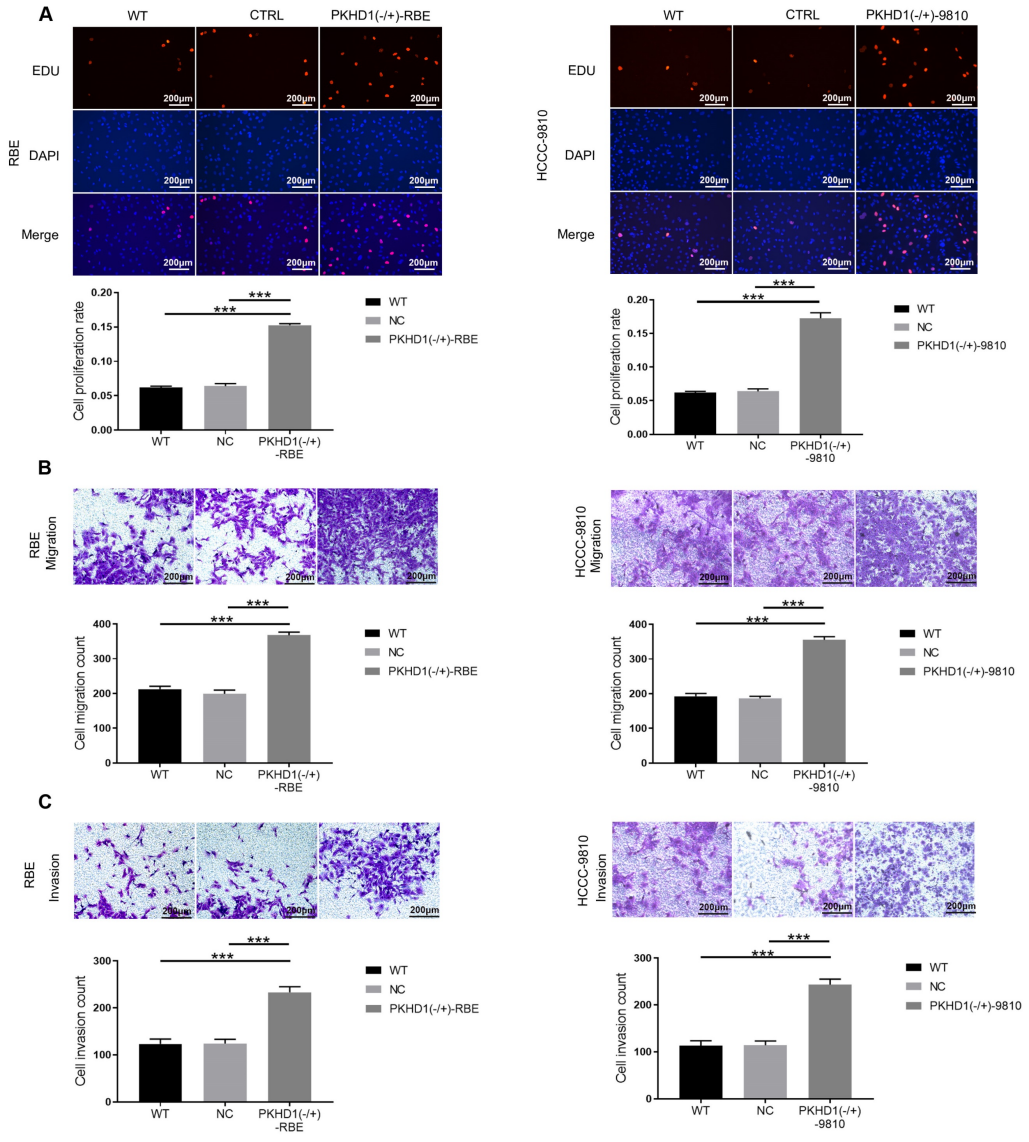
The proliferation, migration, and invasion assays of mixed-clones (***, *P* < 0.001). (A) Cell proliferation was determined by Edu assay; (B) Cell invasion was measured using Transwell; (C) Cell migration assays using Transwell.

Compared with the CTRL and WT groups, the cell proliferation rate in the PKHD1(-/+) group was significantly higher (*Figure 2A*, *P*<0.001). Moreover, transwell assays showed that the cell migration and invasion of PKHD1(-/+) cells were markedly promoted relative to those in the CTRL and WT group (*P*<0.001, *Figure 2B-C*). Collectively, the results demonstrated that the knockdown of PKHD1 enhances cell proliferation, migration, and invasion of ICC cells.

### 3.4. Knockdown of PKHD1 activates the Notch pathway

**Figure 3:**
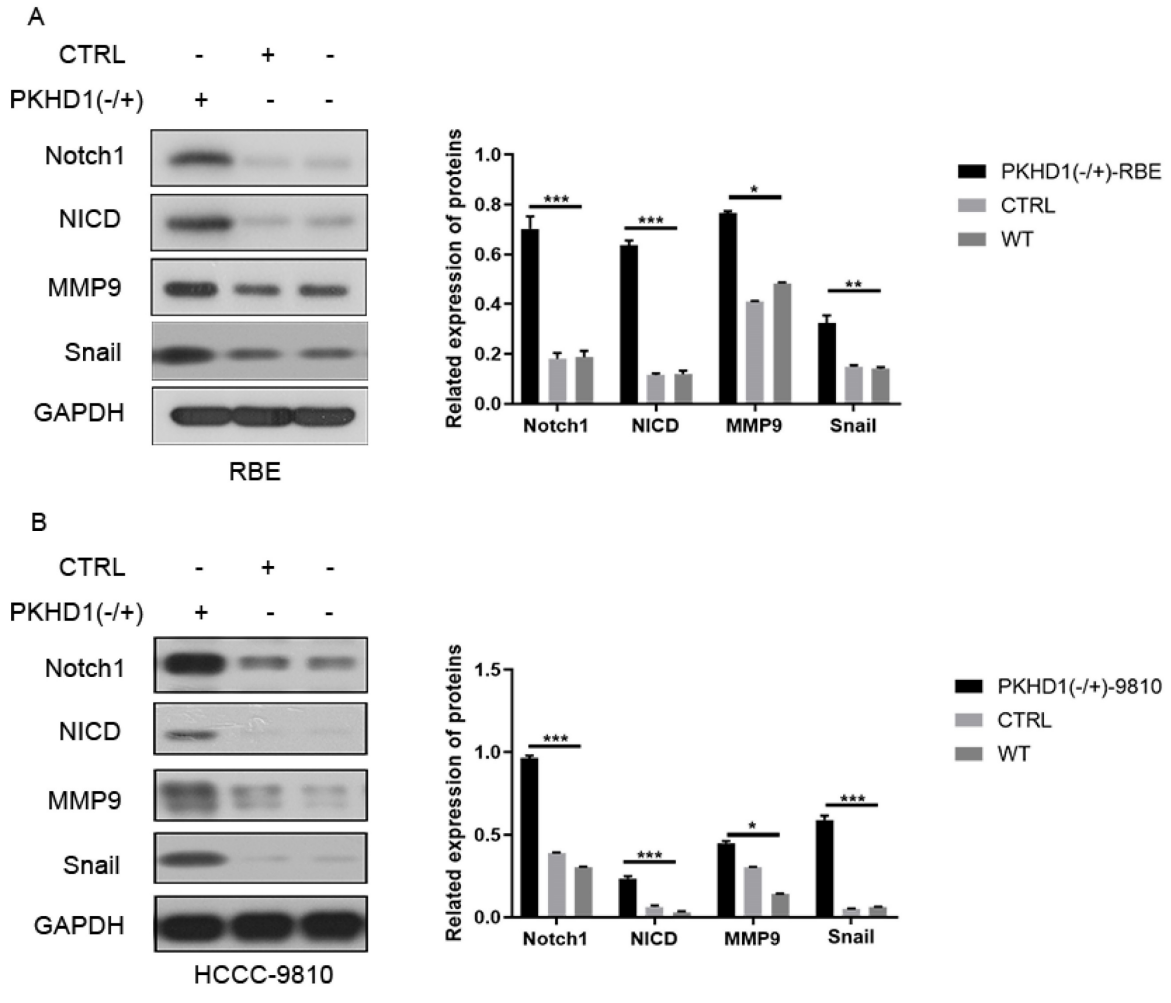
The expressions of Notch pathway and epithelial-mesenchymal transition-related protein in mixed-clones (***, *P* < 0.001).

The results of immunofluorescence colocalization of PKHD1 protein and Notch1 protein are shown in *Figure 1E*. Western blotting suggested that the expressions of Notch1 pathway-related proteins and EMT-related proteins significantly increased in the PKHD1(-/+) group compared to that in the CTRL group (*Figure 3A-B*). These results indicated that the knockdown of PKHD1 may activate the Notch1 pathway.

### 3.5. Using the antagonist of the Notch1 signaling pathway to rescue *in vitro*

**Figure 4:**
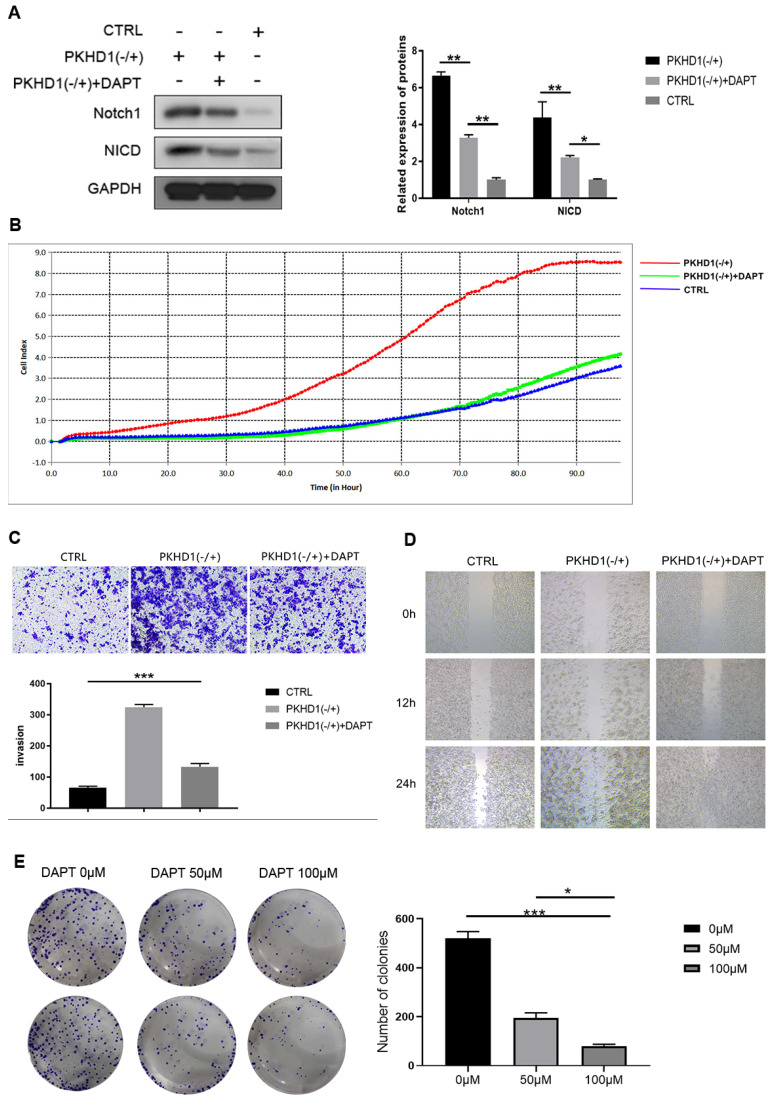
The rescue experiments *in vitro*. (A) Verifying of expression of Notch1 and NICD after adding DAPT in CTRL and PKHD1(-/+) by western blotting; (B) Cell proliferation was determined by RTCA assay;(C) Cell invasion was measured using Transwell; (D) Cell migration assays using scratch assay. (E) Colony formation assays were performed to detect the effects of DAPT on the toxicity and colony formation ability of RBE cells.

DAPT, an antagonist of the Notch1 signaling pathway, was used in the rescue experiment *in vitro*. As exhibited in *Figure 4A*, DAPT inhibited the expression of Notch1 and NICD and partially counteracted the negative-regulated effects of PKHD1(-/+) on proliferation, migration, and invasion of RBE cells (*Figure 4B-D*). Furthermore, we found the colony formation ability of cells was DAPT dose-dependent, but the cytotoxicity of DAPT did not affect the size of the colonies (*Figure 4E*).

### 3.6. *In vivo* study

**Figure 5:**
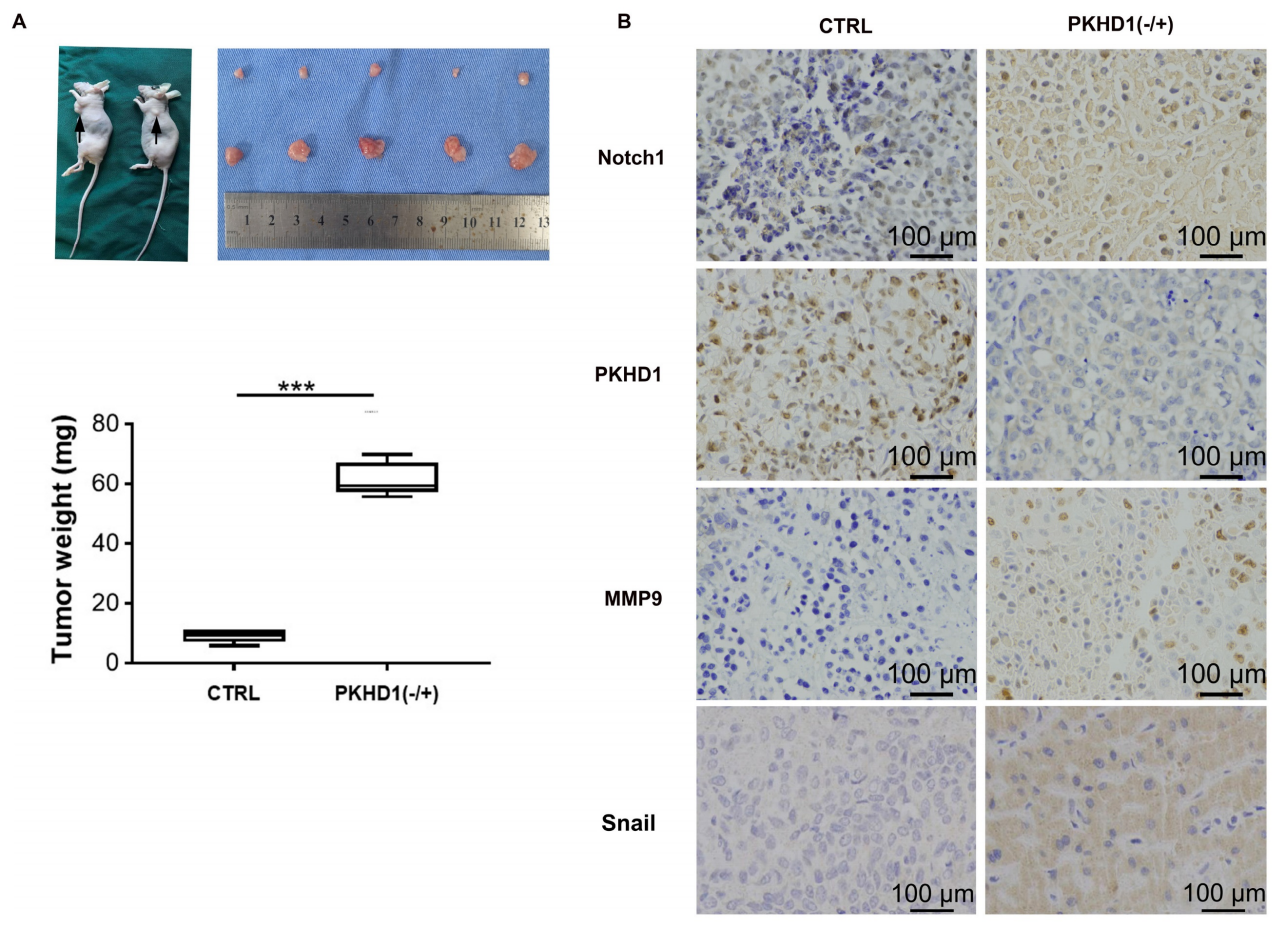
Investigating the effects of PKHD1 on the malignant biological behaviors of ICC cells by subcutaneous tumor formation experiments in nude mice. (A) Subcutaneous tumor formation and tumor weight statistics in nude mice (***, *P* < 0.001); (B) Immunohistochemistry of MMP9 and Snail in the PKHD1(-/+) and CTRL group.

The results of subcutaneous tumor implantation in nude mice suggested that the growth rate, volume, and weight of tumors in the PKHD1(-/+) group was significantly increased compared to the CTRL group (*Figure 5A, P*< 0.001). This indicated that the knockdown of PKHD1 might promote the tumorigenesis ability of ICC cells. Moreover, the results of immunohistochemistry showed that the expression of MMP9 and snail, which was closely related to EMT, was increased after PKHD1 was knocked down (*Figure 5B*).

## 4. Discussion

In this study, the expression of PKHD1 was found to be significantly decreased in human cholangiocarcinoma tissues. Down-regulating the expression of PKHD1 could promote the proliferation and invasion of ICC via activating the Notch pathway.

PKHD1 gene encodes FPC protein, which is a large type I transmembrane protein located in the primary cilia^14^. FPC protein not only acts as a membrane-bound receptor but also regulates cell morphogenesis, adhesion, and proliferation^15^, ^16^. It was reported that PKHD1 mutations may be related to the Von Meyenburg complex, which has the potential to develop into ICC^17^, ^18^. Besides, PKHD1 was identified as a possible candidate gene for colorectal cancer and may be a risk factor for peritoneal metastasis^19^, ^20^. In this study, we investigated the malignant biological behavior changes of ICC cells after the knockdown of the PKHD1 gene, indicating the PKHD1 gene might inhibit the proliferation, migration, and invasion of ICC cells.

The regulatory mechanisms of the PKHD1 gene in malignancy have not been fully elucidated. FPC protein and Notch receptor are both located on the surface of the bile duct cells and are involved in the development and disease process of the bile duct. In this study, we demonstrated that the Notch1 and NICD were up-regulated, suggesting that down-regulating the expression of PKHD1 might activate the Notch pathway.

The Notch pathway is a signaling pathway that involves four Notch receptors (Notch1-4) and five ligands (Jagged1-2, Dll1, Dll3, and Dll4) in mammals^21^, ^22^. The activation of the Notch pathway leads to the release of the Notch intracellular domain (NICD), which subsequently activates the transcription of the target gene^22^. It is reported that the Notch pathway plays an important role in regulating homeostasis, cell proliferation, differentiation, and apoptosis^23^, ^24^. The Notch pathway is also involved in the proliferation and migration of ICC cells as previously reported^25^-^27^. This study found an up-regulated expression of the Notch pathway and the downstream proteins after the knockdown of the PKHD1 gene in ICC cells. Moreover, the malignant phenotype of ICC cells was rescued after the addition of Notch1 inhibitor DAPT, suggesting the protective effects of PKHD1 on ICC were Notch-pathway-dependent.

The Notch pathway is closely related to tumor proliferation, invasion, and metastasis, and one of its possible mechanisms is to induce EMT^28^. It is reported that EMT is related to the immune escape of ICC cells and is an effective enhancer of ICC invasiveness^29^. In this study, immunohistochemistry was used to detect the expressions of EMT-related protein MMP9 and Snail in the PKHD1(-/+) and the normal control tumor-bearing mice. The results further verified that the enhanced malignant biological behavior of ICC cells caused by the knockdown of PKHD1 might be related to the activation of the Notch pathway.

## 5. Conclusion

In summary, this study demonstrated that PKHD1 inhibits the proliferation, migration, and invasion of ICC, and the activation of the Notch pathway might be the downstream mechanism of the protective effects of PKHD1 during the progression of ICC. This suggested that PKHD1 might be a potential therapeutic target for the treatment of ICC. In addition, the expression of PKHD1 in human ICC tissues was significantly decreased, indicating that it could be a feasible biomarker for the early diagnosis of ICC.

## Supplementary Material

Supplementary table.

## Abbreviations

RTCA: Real-Time Cellular Analysis
EMT: epithelial-mesenchymal transition
FPC: fibrocystin/polyductin
ICC: intrahepatic cholangiocarcinoma
NICD: Notch intracellular domain
PKHD1: polycystic kidney and hepatic disease 1
